# Diabetic Macular Oedema Guidelines: An Australian Perspective

**DOI:** 10.1155/2023/6329819

**Published:** 2023-02-14

**Authors:** Yew Sen Yuen, Jagjit Singh Gilhotra, Michelle Dalton, Jaskirat S. Aujla, Hemal Mehta, Sanj Wickremasinghe, Gurmit Uppal, Jennifer Arnold, Fred Chen, Andrew Chang, Samantha Fraser-Bell, Lyndell Lim, Janika Shah, Ellie Bowditch, Geoffrey K. Broadhead

**Affiliations:** ^1^National University Hospital, Singapore; ^2^Royal Adelaide Hospital, Adelaide, Australia; ^3^Dalton & Associates Inc., Pennsylvania, USA; ^4^South Australian Institute of Ophthalmology, Adelaide, SA, Australia; ^5^Save Sight Registries, University of Sydney, Sydney, NSW, Australia; ^6^Strathfield Retina Clinic, Sydney, Australia; ^7^Centre for Eye Research Australia, The Royal Victorian Eye and Ear Hospital, University of Melbourne, Melbourne, Australia; ^8^Moreton Eye Group, Brisbane, Queensland, Australia; ^9^Marsden Eye Specialists, Sydney, NSW, Australia; ^10^Centre for Ophthalmology and Visual Sciences (Incorporating Lions Eye Institute), The University of Western Australia, Nedlands, WA, Australia; ^11^Ophthalmology, Department of Surgery, University of Melbourne, Victoria, Australia; ^12^Sydney Institute of Vision Science, University of Sydney, Sydney, NSW, Australia; ^13^Sydney Retina Clinic and Day Surgery, University of Sydney, Sydney, NSW, Australia; ^14^Save Sight Institute, University of Sydney, Sydney, NSW, Australia; ^15^Department of Ophthalmology, Save Sight Institute, University of Sydney, Sydney, NSW, Australia; ^16^Sydney Eye Hospital, Sydney, Australia; ^17^Singapore National Eye Centre, Singapore

## Abstract

The number of people living with diabetes is expected to rise to 578 million by 2030 and to 700 million by 2045, exacting a severe socioeconomic burden on healthcare systems around the globe. This is also reflected in the increasing numbers of people with ocular complications of diabetes (namely, diabetic macular oedema (DMO) and diabetic retinopathy (DR)). In one study examining the global prevalence of DR, 35% of people with diabetes had some form of DR, 7% had PDR, 7% had DMO, and 10% were affected by these vision-threatening stages. In many regions of the world (Australia included), DR is one of the top three leading causes of vision loss amongst working age adults (20–74 years). In the management of DMO, the landmark ETDRS study demonstrated that moderate visual loss, defined as doubling of the visual angle, can be reduced by 50% or more by focal/grid laser photocoagulation. However, over the last 20 years, antivascular endothelial growth factor (VEGF) and corticosteroid therapies have emerged as alternative options for the management of DMO and provided patients with choices that have higher chances of improving vision than laser alone. In Australia, since the 2008 NHMRC guidelines, there have been significant developments in both the treatment options and treatment schedules for DMO. This working group was therefore assembled to review and address the current management options available in Australia.

## 1. Introduction

In 2015, the Global Burden of Disease report estimated that the prevalence of diabetes rose from approximately 333 million people in 2005 to approximately 435 million people in 2015, an increase of 30.6% [1], underscoring the increasing burden of this disease. Furthermore, the number of people living with diabetes is expected to rise to 578 million by 2030 and to 700 million by 2045 [2], exacting a severe socioeconomic burden on healthcare systems around the globe. This is also reflected in the increasing numbers of people with ocular complications of diabetes (namely, diabetic macular oedema (DMO) and diabetic retinopathy (DR)) [3]. In one study examining the global prevalence of DR [4], 35% of people with diabetes had some form of DR, 7% had PDR, 7% had DMO, and 10% were affected by vision-threateningDR. The International Diabetes Federation's prevalence rates are higher, with an estimated annual incidence of DR ranging from 2.2% to 12.7% and an annual progression to sight-threatening DR ranging from 3.4% to 12.3% [2, 5]. In many regions of the world (Australia included), DR is one of the top three leading causes of vision loss amongst working age adults (20–74 years) [6].

In the management of DMO, the landmark ETDRS study [7] demonstrated that moderate visual loss, defined as doubling of the visual angle, can be reduced by 50% or more by focal/grid laser photocoagulation. However, over the last 20 years, anti-VEGF [8–10] and corticosteroid therapies [11] have emerged as alternative options for the management of DMO and provided patients with choices that have higher chances of improving vision than laser alone. In Australia, since the 2008 NHMRC guidelines, there have been significant developments in both the treatment options and treatment schedules for DMO. This working group was therefore assembled to review and address the current management options available in Australia.

### 1.1. Search Strategy

The group chair used both Medline and PubMed to retrieve relevant literature up to 2019, which was supplemented by the individual authors using search terms relevant to the subject matter covered in each section. The latest UK guidelines [12], the American Academy of Ophthalmology's Preferred Practice Patterns [13], RANZCO and NHMRC position statements [14, 15], the Royal College of Ophthalmologists DR Guidelines [16], and the European Retina Guidelines [17] were used as reference sources. There was no plan to include economic data in this review, and no cost analysis was incorporated.

## 2. Epidemiology of DMO and Related Vision Loss

Globally, an aging global population is expected to spur an increase in the number of patients with diabetes. Besides an aging population [18, 19], rising obesity rates [18] and decreasing mortality rates [20] have also contributed to the increasing prevalence of diabetes. For more than 2 decades, diabetes has been recognised as a chronic, debilitating, and costly disease [21].

In Australia, it is estimated that, unless trends in diabetes incidence are reversed, there will be at least 2 million Australian adults with diabetes by 2025. If instead, obesity trends, and consequently diabetes incidence trends, continue upwards, and mortality continues to decline, the projections predict that around 2.5–3 million people will have diabetes by 2025 with the figure closer to 3.5 million by 2033 [22]. This will lead to a significant increase in the health impact and economic burden of DR [23]. Optometry Australia notes that the proportion of people with DM in the Indigenous population is consistently three times greater than in the non-Indigenous population [24].

In two landmark, subnational, population-based studies conducted in the early 1990s (the Melbourne Visual Impairment Project (VIP) [25] and the Blue Mountains Eye Study [26]), the prevalence of DR in non-Indigenous Australian adults was reported as 29.1% and 32.4%, respectively. The 2017 National Eye Health Survey (NEHS) reported the prevalence of DR amongst non-Indigenous and Indigenous Australian adults who self-reported diabetes as 28.5% and 39.4%, respectively [27].

Looking specifically at DMO, prevalence ranges from 3.3% to 6.0% as reported in Australian patients with diabetes [23, 27]. Furthermore, the 2017 NEHS survey reported DMO as one of the top reasons for vision loss in Australia, especially amongst Indigenous Australians, with most of those diagnosed with DMO reported to have sight-threatening DR [28, 29].

## 3. Pathophysiology of DMO

DR is caused by elevated blood glucose levels that gradually damage small blood vessels within the retina, and DR is closely related to the duration of diabetes in conjunction with a lack of systemic control of the disease [30–33]. In healthy eyes, retinal capillaries are nonfenestrated and the capillary endothelial cells have tight junctions preventing leakage. In diabetic eyes, however, the presence of retinal pathology may lead to fluid leakage that, in turn, causes oedema or swelling.

The typical features of DR can also be explained by the “neurovascular unit” concept [34] which refers to the intricate functional coupling between neurons, glial cells, and blood cells. These cells work in close coordination to integrate retinal vascular flow with metabolic activity, and it has been shown that all components of the neurovascular unit are disrupted by diabetes, leading to loss of autoregulation and the typical clinical features of diabetic retinopathy.

DMO is secondary to the retinal barrier rupture and a consequence of DR. The landmark Diabetes Control and Complications Trial (DCCT) found that 27% of people with type 1 diabetes will develop DMO within 9 years of disease onset, while the Wisconsin Epidemiologic Study of Diabetic Retinopathy (WESDR) found that the incidence of DMO in people with type 2 diabetes differed in those who needed insulin (25%) and those who did not (14%) [35–37].

### 3.1. Clinical and Morphological Features

DR causes vascular changes including microaneurysms, exudates, haemorrhages, and, in severe cases, neovascularisation.

The breakdown of the blood-retinal barrier results in increased permeability of the retinal vasculature [38] and leads to DMO. Vascular leakage through the endothelial and paracellular routes is the hallmark of DMO. When macula thickening involves the fovea, metamorphopsia and vision disturbance can follow [35]. There remains debate on the role of choroidal thickness in the onset of clinical DR [12, 39, 40].

### 3.2. The Role of Inflammation

Inflammation is considered an intrinsic feature of systemic diabetes/insulin resistance that involves the release of cytokines from adipose tissue which then impairs insulin action, affecting people with either type of diabetes [41]. It is now readily accepted that inflammation is a key component in the pathogenesis of DR, as borne out by diffuse vascular dilation, tortuosity, leakage and neovascularisation, atrophy of the retinal neural parenchyma, macular oedema, and fibrosis; however, the relationship of systemic inflammation with DR remains unclear [41]. The late phase of DMO may be less driven by angiogenic mediators than by inflammatory mediators [42].

A variety of biochemical factors with proinflammatory and/or vascular effects, such as angiopoietin-2, chemokine ligand-2, intercellular adhesion molecule-1 (ICAM-1), interleukin 6, monocyte chemotactic protein-1 (MCP-1), tumour necrosis factor (TNF), vascular cell adhesion molecule 1 (VCAM-1), and VEGF, have been implicated in DMO [43, 44]. Only two of these factors, VEGF and MCP-1, are inhibited by currently available anti-VEGF treatments, whereas all these factors are inhibited by corticosteroids [45].

## 4. Diabetic Macular Oedema Screening

RANZCO, the NHMRC, and Optometry Australia have provided guidance on when to refer patients with diabetes to ophthalmologists for retinal screening. However, it is reported that fewer than 50% of Australians receive appropriate screening [46–48]. Those in remote or rural communities may be further disadvantaged by not having access to ophthalmic services. Blindness from ocular diseases is six times higher in Indigenous Australians than in non-Indigenous people, yet <20% receive DR screening, and more than 35% have never been screened [48]. DR affects 5.2% of Aboriginal and Torres Strait Islander populations compared to just 1.4% of non-Indigenous Australians [29, 46]. Furthermore, only half of all Aboriginal and Torres Strait Islanders with diabetes undergo annual eye exams for DR, and up to 25% will not have a screening test at all; comparatively, 78% of non-Indigenous adults undergo a screening test yearly [15].

RANZCO updated its guidelines in 2020, and the NHMRC has not updated its guidelines since 2008. Herein, we will review the recommendations and guidelines and offer our real-world clinical perspectives. There remains a recognised need that eye care professionals and primary healthcare centres will need to increase remote screening to keep up with the increasing numbers of patients with diabetes and diabetic eye disease.

In general, testing is recommended semiannually for those with well-controlled systemic disease and quarterly for those without well-controlled disease; those with no evidence of DR and well-controlled glycaemia can be screened every 1-2 years [49].

### 4.1. RANZCO

RANZCO acknowledges that DR screening is likely to be performed by optometrists and ophthalmologists, but it also notes that other healthcare professionals with adequate training may perform DR screening tests as part of an overall comprehensive diabetes care regimen [15]. GPs, in particular, can screen for DR in more rural and regional areas. RANZCO follows the NHMRC's 2008 recommendations [14] that all patients with DM undergo DR screening at the time of diagnosis and then every 2 years if no retinopathy is detected.

As failure to attend DR screening exams represents a modifiable risk factor for vision loss [14], RANZCO recommends determining the date of the most recent screening. RANZCO recommends the following screening and referral pathways for optometrists:

- Patient presents/screened for DR at presentation (children screened at puberty)
- Clinical assessment
- Grade VA (6/12 or worse should be referred; 6/9 to 6/12 should be considered for referral)
- Grade image quality (if inadequate after repeated imaging, refer to an ophthalmologist)
- Grade DR
- Communicate results within 2 weeks to GPs, endocrinologists, and ophthalmologists

RANZCO has published a set of clinical notes to accompany its recommendations for screening and referral. One key clinical modifier component is that patients at higher risk should be evaluated annually regardless of evidence of DR on examination. Clinical modifiers include duration of diabetes >15 years, suboptimal glycaemic control (A1c >8% or 64 mmol/mol), systemic disease, including poorly controlled hypertension or abnormal serum lipids, or other diabetic complications, pregnancy, and Aboriginal and Torres Strait Islanders and overseas-born Australians from South Pacific, the Middle East, North Africa, Southern Asia, and Southern Europe.

Anyone who screens and/or grades DR can complete an online DR grading course to ensure familiarity with the clinical features of DR/DMO. RANZCO recommends the University of Melbourne Self-Directed Retinopathy Grading Course (http://drgrading.iehu.unimelb.edu.au/cera/index.asp).

### 4.2. NHMRC

The NHMRC guidelines also recommend annual retinal screening and VA assessment for Indigenous Australians and biennial assessment for non-Indigenous Australians with diabetes but without significant risk factors for DR [14]. As noted previously, these guidelines were issued in 2008 and have not been updated; the newer RANZCO guidelines use the NHMRC as their basis, and as such, a thorough review of the NHMRC screening guidelines will not be reiterated.  Table 1 summarises the recommendations for special groups. The guidelines do recommend that exams be increased in frequency (to as many as 4 times annually) if NPDR is detected and that referral to an ophthalmologist within 4 weeks be considered when DMO or PDR is suspected, or when unexplained changes in vision occur.

Foreman et al. [46] examined the adherence to these guidelines as a part of the National Eye Health Survey, separating results into Indigenous (aged 40 years or more) and non-Indigenous (aged 50 years or more) Australians. The findings suggest that an integrated DR screening service is needed, particularly in more remote areas, to improve adherence: 77.5% of non-Indigenous Australians adhere to the guidelines, but only 52.7% of Indigenous Australians do (*P* < 0.001). Furthermore, 26.2% of Indigenous Australians with diabetes reported that they had never undergone a diabetic eye examination, compared with 15.3% of non-Indigenous participants (*P* < 0.001). Almost three quarters of both groups said they were unaware of the need for regular eye exams.

The adherence rate reported by Foreman et al. for non-Indigenous Australians is similar to the 77% reported in the AusDiab study, [51] but that study included a much broader range of participants (25 years and older). Foreman et al. instead compared their results to those of the Melbourne Vision Impairment Project, which found an adherence rate in 1998 for only 50% of non-Indigenous Australians [52]. The authors suggested the improved adherence rates—while still needing vast improvement—may be due to increased access to DR screening services and/or increased knowledge about the need for screening amongst healthcare providers.

### 4.3. Other Guidelines

Beyond the Australian guidelines discussed above, both the AAO and the UK Consensus Working Group have published guidelines on DR [12, 13].

AAO [13] recommends that screening begin 5 years after a diagnosis of type 1 DM and then annually, as “substantial retinopathy” may develop within 6-7 years of onset. Patients with type 2 DM should be referred for ophthalmology assessment upon diagnosis. In the UK, screening is considered a public health programme and everyone with diabetes is screened for DR, with an age for entry into the UK DR screening programmes set at 12 years old [12]. The National Screening Committee [53] updated its classification of DR in screening as follows:

- Absence of DR: R0
- Presence of microaneurysms and small retinal haemorrhages or mild, non‐PDR: R1
- Moderate to severe NPDR: R2
- PDR: R3
- No diabetic maculopathy: M0
- Suspected diabetic maculopathy: M1

Urgent referral to an ophthalmologist within 2 weeks is recommended for PDR (R3). As per the UK Consensus Working Group, the successful screening programme has led to a reduction in blindness from a diabetic eye disease such that it is no longer the most common cause of blind certification in the working age group [12, 54]. The group adds that using OCT in addition to photographic screening can help improve the sensitivity and specificity of early diagnosis of DMO [12].

## 5. Diagnosis and Classification of DMO

### 5.1. Standard Imaging

Initial visual examinations should include VA, slit-lamp biomicroscopy, IOP, gonioscopy (before dilation, when indicated), pupillary assessment for optic nerve dysfunction, fundoscopy (including stereoscopic examination of the posterior pole), and an examination of the peripheral retina and vitreous [13].

Ancillary tests may provide clinicians with more detailed information that may enhance patient care; these tests may include color and red-free fundus photography, OCT, fluorescein angiography (FA), OCT-angiography (OCTA), and B-scan ultrasonography [13]. To date, OCT is considered the most effective tool for diagnosing DMO: it is able to identify anatomic/structural changes in the retina caused by DMO that other modalities cannot and is considered more objective than stereoscopic photographs or clinical exams [3, 12, 13].

FA is considered a useful diagnostic tool to help differentiate diabetic macular swelling from other macular diseases, and advances in widefield FA have shown improved detection of peripheral ischemia and peripheral lesions that may not be clinically apparent [13]. B-scan ultrasonography plays an important role in the assessment of vitreoretinal traction and tractional detachment in the macular region in severe diabetic eye disease with media opacity [55].

#### 5.1.1. Advanced Imaging

*(1) OCT*. OCT was first introduced in the 1990s but did not become a standard of care for macular disease until 2005. While RANZCO does not mandate OCT as a part of routine screening [56], we feel that it should be offered as a part of screening wherever available to help detect the presence of centre-involving DMO.

Most guidelines [12–14, 56] recommend using OCT to evaluate DR and DMO, especially in the following scenarios:

- When there is unexplained vision loss
- Detect, quantify, and monitor DMO
- Identify areas of vitreomacular traction
- Evaluate patients with difficult or questionable exams for DMO

*(2) OCTA*. OCTA, introduced in 2014, is a noninvasive modality that generates volumetric angiography images (without the use of dye) within seconds that has the “clinical capability of specifically localising and delineating pathology along with the ability to show both structural and blood flow information in tandem” [57]. OCTA may provide additional information to determine the presence of ischemic maculopathy in diabetic patients, demonstrated by the presence of an enlarged foveal avascular zone (FAZ) [58]. OCTA may be better than FA to define the central and parafoveal macular microvasculature and delineate FAZ because it is not covered by fluorescein from dye leakage [59]. The superficial and deep capillary plexuses are noted to have a larger FAZ using OCTA [60]. Of note, FAZ measurements cannot be compared using different OCT devices, but repeating measurements with the same device would allow repeated, reliable measurements of the FAZ [61]. Image magnification adjustment to axial length variation has been recommended prior to interindividual comparison [62].

AAO notes that the guidelines and indications for use of OCTA in the US during screening and management of DR are still evolving [13].

### 5.2. Ultrawide Field Imaging

The most recent RANZCO guidelines allow the use of an ultrawide field (UWF) image as an alternative to the more traditional method of two 45-degree fundus images centred on the macula and nasal fundus [56, 63, 64]. UWF is defined by images showing retinal features anterior to the vortex vein ampullae in all four quadrants [65]. Several studies have demonstrated significant differences between UWF imaging devices in the area of retinal image capture, DR lesion detection rates, and DR grading [66–69].

### 5.3. Classification

Two major, well-accepted grading systems exist that classify stages of DR and have been used for decades. These are the International Clinical Diabetic Retinopathy and Diabetic Macular Oedema Disease Severity Scale [70] and the Wisconsin System (modified Airlie House classification) [71–73]. Both of these grading systems are also cited in the National Health and Medical Research Council (NHMRC) guidelines on the assessment of diabetic retinopathy [27]. There are five levels for grading DR based on the risk of progression:

1. None
2. Mild NPDR
3. Moderate NPDR
4. Severe NPDR
5. PDR (in the presence or absence of DMO)

DMO can be diagnosed by the presence of clinically significant macular oedema as defined by the landmark 1985 ETDRS study [7]. This was defined as follows:

- Retinal oedema within 500 *μ*m of the fovea
- Hard exudates within 500 *μ*m of the fovea with retinal oedema
- Retinal oedema of 1500 *μ*m in size within 1500 *μ*m of the fovea

However, the more recent landmark DRCR trials have focused on the presence of central-involved DMO defined by an OCT central subfoveal thickness of 250 *μ*m or more on Zeiss Stratus or the equivalent on spectral-domain OCTs based on gender-specific cutoffs [74]. The newer generations of spectral-domain and swept-source OCT are able to identify more qualitative features in better detail than the Zeiss Stratus time-domain OCT. These include subretinal fluid, intraretinal cystoid fluid, disorganization of retinal inner layers, and the status of the vitreomacular interface which have varying prognostic factors for the visual outcome and response to treatment.

## 6. Medical Management of DM

DM is characterised by hyperglycaemia and is caused by defects in insulin secretion, insulin action, or both; its varied, biochemical, and clinical manifestations render it one of the most common metabolic diseases in humans [75]. Although several types are currently recognised, type 1 and type 2 are the most common. Cellular-mediated autoimmune destruction of pancreatic beta cells causes an absolute deficiency of endogenous insulin, resulting in type 1 DM. Type 2, however, comprises more than 95% of the global diabetic population and is caused by an insulin deficiency, and while people with type 2 DM can secrete insulin, they cannot secrete enough to overcome insulin resistance [75].

Typically, diagnosis is based on glucose tolerance: normal glucose homeostasis, impaired glucose homeostasis, or DM. Glucose tolerance can be assessed by fasting plasma glucose (FPG), the response to oral glucose challenge, or the haemoglobin A1c (A1c) test [76]. Worsening glucose homeostasis occurs, followed by the development of hyperglycaemia [76].  Table 2 shows the spectrum from normal glucose tolerance to DM; the arrows suggest changes in glucose tolerance can be bidirectional (i.e., someone with type 2 diabetes may revert to impaired glucose tolerance after weight loss).

Signs and symptoms vary widely, as does the severity of hyperglycaemia, but it is well accepted that microvascular and macrovascular damage and complications are dependent on the degree and duration of hyperglycaemia. Chronic hyperglycaemia is associated with complications ranging from long-term damage to failure of various organs including the eyes, blood vessels, heart, nerves, and kidneys [75].

### 6.1. Managing and Monitoring DM

Multiple therapeutic approaches have been developed for various aetiologies. Traditionally, the primary goal of managing DM is to emphasise blood glucose control, and monitoring A1c is recognised as a key component to achieving that goal. Likewise, reducing or eliminating the complications of DM is a key component. Both of these goals should be individualized for each patient [15, 77]. RANZCO notes that lifestyle changes in people with prediabetes have resulted in a reduction of healthcare costs of about $1087 per person in a lifetime [15].

Although treatment and management of DM are multidisciplinary, patient buy-in and involvement are of paramount importance. The ADA, RANZCO, and the European Association for the Study of Diabetes consensus report recommend a patient-centred approach to choose the appropriate pharmacologic treatment [15, 49]. The comprehensive care of the patient with DM includes an emphasis on nutrition, exercise, and proper self-monitoring of blood glucose coupled with in-office A1c testing. The ADA suggests that the goal is to achieve an A1c level as close to normal as possible without significant hypoglycaemia (which is typically a level of <7% and for some, ≤6.5%) [77]. Recommendations are based on data from several large population-based studies that found glycaemic control is the cornerstone to systemic disease control and diabetic complications including DR and DMO [33, 77–84]. An increasing awareness of retinopathy risk, coupled with earlier identification and treatment for people with DR as well as improved medical management of glucose, blood pressure [84–93], and serum lipids [94–96], has resulted in a reduced rate of incidence and prevalence since 1985 [97], yet the prevalence is still expected to double in the next 20 years.

### 6.2. Pharmacologic Approaches for Managing DM

Pharmacologic approaches typically begin with glucose-lowering (oral) agents initially, but since type 2 DM is considered a progressive disorder, multiple therapeutic agents and (potentially) insulin may be needed [77].  Table 3 describes common pharmacologic classes used in the treatment of DM coupled with their side effects. Optimising blood pressure and serum lipid control is recommended to reduce the risk of DR or to slow its progression [49].

#### 6.2.1. Systemic Treatments and DR

Chronic hyperglycaemia, nephropathy, hypertension, and dyslipidaemia are additional factors that increase the risk of DR; intensive diabetes management has also been shown in large, prospective studies to slow the onset and progression of DR [49].

For patients with dyslipidaemia, fenofibrate has been shown to slow DR progression, particularly in patients with very mild NPDR [49, 96, 103]. The ACCORD study (*N* = 10251) showed that intensified management of glycaemia significantly reduced diabetic eye outcomes compared to standard therapy for 3-line change in visual acuity at transition (HR 0.91, CI 0.84–0.98; *P* = 0.012) and at the end of the study (HR 0.94, CI 0.89–1.00; *P* = 0.05) [96, 104]. Cataract extraction was also significantly reduced in the intensive group compared to the standard group at end of the study (HR = 0.89, CI 0.80–0.99; *P* = 0.026) [105]. However, after 10 years' duration, fenofibrate benefit regressed; intensive BP control had no effect [104]. The FIELD study (*N* = 9795) showed that treatment with fenofibrate resulted in a statistically significant relative reduction for laser treatments (37%; *P* = 0.0003) after an average of 5 years' treatment with fenofibrate [95, 106]. In a substudy of FIELD [106] (*n* = 1012), a 2-step progression of retinopathy grade did not differ significantly between patients on fenofibrate (*n* = 46 (9.6%)) or the placebo (*n* = 57 (12.3%); *p* = 0.19) or in the subset of patients without pre-existing retinopathy (*n* = 43 (11.4%) in the fenofibrate group vs. 43 (11.7%) in the placebo group; *P* = 0.87). By contrast, in patients with pre-existing retinopathy, significantly fewer patients on fenofibrate had a 2-step progression than did those on the placebo (3 (3.1%) patients vs. 14 (14.6%), respectively; *P* = 0.004). In PDR, the risk reduction was 30% (*P* = 0.015), corresponding to an absolute risk reduction of 0.7%. In 2013, Australia became the first country in the world to recommend fenofibrate to slow DR progression [107]. The Diabetic Retinopathy Clinical Retina Network (DRCR.net) is currently undertaking a study on the use of fenofibrate (Protocol AF) on the management of DR (https://public.jaeb.org/drcrnet/stdy).

Section 7 discusses the ophthalmic management of DMO.

## 7. Ophthalmic Management of DMO

In today's clinical setting, treating and managing patients with DMO are often determined by clinical findings on OCT [13], which allows for milder degrees of oedema to be detected and/or treated than earlier technologies (i.e., slit lamp). However, it remains important to quantify the amount of centre-involving DMO (CI-DMO) before determining a treatment regimen.

As noted earlier in this paper, studies consistently show that systemic glycaemic control and/or intensive glycaemic therapy remain a valuable and primary component to avoid ocular complications and/or vision loss [5, 96, 108–110]. The ACCORD study—considered a hallmark study when it was published more than a decade ago—showed that vision loss (3-line change) was smaller in the intensive treatment group than in the standard therapy group [96, 105]. Other conditions (including sleep apnoea) may cause or exacerbate macular oedema in people with type II diabetes [111–113].

In both Europe and the United States, treatment options include intravitreal therapies with either intravitreal anti-VEGF injections or corticosteroid implants (dexamethasone or fluocinolone implants or triamcinolone) [17]. In Australia, current treatment options include first-line intravitreal therapies with anti-VEGF drugs, with second-line treatments including laser therapy or steroids (dexamethasone implants and triamcinolone (off-label)) [17].

### 7.1. Anti-VEGF

The use of intravitreal anti-VEGF remains the mainstay of therapy for CI-DMO. Several large RCTs provide the rationale for use of anti-VEGFs in DMO, amongst them, RIDE and RISE, RESTORE, VIVID and VISTA, and the DRCR.net's Protocol T [8, 74, 114–119]. In Australia, three anti-VEGF options are available (aflibercept, bevacizumab, and ranibizumab). More recently, faricimab (Vabysmo), a bispecific antibody that targets both angiopoietin-2 and VEGF-A, was shown to be noninferior to aflibercept every 8 weeks when used in a personalised treatment interval regime [120]. This has been approved by the U.S. Food and Drug Administration and recently in Australia.

The debate amongst clinicians about the appropriate time to switch therapy when patients have a suboptimal or inadequate response is ongoing. Switching amongst agents may be effective: one study showed that aflibercept and ranibizumab may be effective treatments for patients who did not respond to bevacizumab [121]. One recent consensus statement [122] on the management of DMO in Asian patients recommends assessing response to therapy immediately after the first anti-VEGF injection. If vision remains poorer than 6/6 with CMT >300 *μ*m and less than 10% change in CMT after the preceding injection, a switch in the choice of intravitreal agents (other anti-VEGF agents or steroids) is recommended.

The most recent NHMRC guidelines were published before the approval of anti-VEGF agents in Australia and therefore cannot recommend a treatment regimen or a switch timeline. Mansour et al. [123] proposed treatment decision factors in cost constraints, adherence, and provider logistics, often recommending laser as a primary therapy when cost is an issue and anti-VEGF agents when those factors are not an issue. RANZCO does not provide guidance on which anti-VEGF therapy should be considered first but does note that bilateral same-day injections are frequently performed worldwide in an effort to reduce the patients' travel burden.

There are some reports in the literature about the adverse impact of switching medications, including that a switch may lead to sustained IOP increases that may need to be treated topically [124].

Overall, however, the relative safety amongst the three primary anti-VEGF agents is about the same [125, 126] with no signals of differences in overall safety in a 2018 Cochrane review [127].

### 7.2. Laser Photocoagulation

In 1985, the ETDRS study [7] found immediate focal photocoagulation reduced the risk of moderate vision loss by about 50% in patients with DMO compared to deferred photocoagulation. At the time, study authors defined CSME as:

- Retinal thickening within 500 *μ*m of the centre of the macula
- Hard exudates within 500 *μ*m of the centre of the macula if associated with thickening of the adjacent retina
- Retinal thickening of >1-disc area in size, any part of which is located within a 1-disc diameter of the centre of the macula

With focal laser, treatment uses 100*μ*m laser burn applied to the areas of focal leakage plus areas of capillary nonperfusion in the paramacular region. Grid laser applies the same 100*μ*m laser but uses a grid pattern on areas of diffuse leak and nonperfusion around the macula. The NHRMC recommends against using grid laser that does not directly treat focal leaks. The group further notes that treatment is generally unlikely to be beneficial in the presence of significant macular ischemia.

However, for patients with centre-involving DMO, intravitreal therapy has the advantage over focal/grid laser in improving vision and is the main modality of treatment.

#### 7.2.1. Laser and Anti-VEGF Therapy

The use of laser in combination with intravitreal anti-VEGF was investigated in DRCR.net's Protocol I [128], which concluded that, at 5 years, eyes receiving initial ranibizumab therapy for centre-involving DMO were likely to have better long-term vision improvements than eyes managed with laser or triamcinolone + laser followed by very deferred ranibizumab for persistent thickening and vision impairment. Focal/grid laser treatment initiated with intravitreal ranibizumab was no better than deferring laser treatment for ≥24 weeks in eyes with centre-involving DMO with vision impairment. While over half of the eyes where laser treatment is deferred may avoid laser for at least 5 years, such eyes may require more injections to achieve these results when following the DRCR protocol. Most eyes treated with ranibizumab and either prompt or deferred laser maintain vision gains obtained by the first year through 5 years with little additional treatment after 3 years.

### 7.3. Corticosteroids

Corticosteroids are well-known anti-inflammatory agents, and their initial use in the treatment of DMO was reserved for those who have not responded to intravitreal anti-VEGF treatment. There are potential advantages to using corticosteroid implants, including both a reduced treatment burden and predictable pharmacokinetics [129]. Primary treatment with corticosteroids may be of particular benefit in pseudophakic eyes, vitrectomised eyes, eyes with DMO undergoing cataract surgery, and eyes with long-standing DMO [129].

It is further well accepted that intravitreal corticosteroid implants are effective in improving VA and reducing macular oedema in patients with DMO [129–148]. To date, there are three potent synthetic corticosteroids used in the treatment of DMO: dexamethasone, fluocinolone acetonide, and triamcinolone acetonide. Of these, triamcinolone acetonide (off-label use) is used in suspension formulation, with effects lasting about 3 months in nonvitrectomised eyes [149], and both dexamethasone and fluocinolone acetonide are used as intravitreal implants [150–152]. To date, there remains a paucity of clinical trial data comparing the long-term safety of corticosteroids in DMO, and the validity of some findings claiming one is “safer” than another has been challenged [153].

Corticosteroid use can be either first- or second-line treatment and may be more appropriate for one or the other in certain patient populations. In 2014, The RANZCO Clinical Practice Guidelines on the use of intravitreal triamcinolone acetonide suggested that its use as a first-line treatment in pseudophakic patients should be considered before other corticosteroid therapies were available [154]. However, Gilles et al. [155] found that the risks of elevated intraocular pressure (IOP) and cataract formation with the use of intravitreal triamcinolone plus focal/grid photocoagulation were substantially higher when traditional photocoagulation was used on its own, even though better visual outcomes were achieved, suggesting that its use may be better suited for second-line or adjunctive therapies.

In general, newer Australian guidelines and recommendations note that some patient groups—pregnant women, children, those with learning difficulties, and people who have had recent thromboembolic events—may benefit from the use of an intravitreal steroid implant as a first-line therapy rather than an anti-VEGF agent. This follows other international guideline recommendations, specifically those recently published by our UK counterparts [12].

In the UK, corticosteroid implants are approved when there has been an insufficient response to noncorticosteroid medical therapy or when treatment with a corticosteroid does not result in a significant rise in IOP [151, 156–158]. Australian authorities have recognised Iluvien (fluocinolone acetonide, Alimera Sciences) as of 2019, but to date, this implant is not often used and will be discussed only in short detail in this guidance.

 
*Ozurdex (Dexamethasone, Allergan Australia Pty. Ltd.)*
  Ozurdex is a biodegradable, sustained-release implant containing 700 *μ*g dexamethasone in a solid polymer drug delivery system. The Ozurdex implant is preloaded into a single-use, specifically designed DDS applicator to facilitate injection of the rod-shaped implant directly into the vitreous. Polymer DDS contains the polyglactin D,L lactide-coglycolide (PLGA) biodegradable polymer matrix. The implant itself is preservative free. Retreatment for responders is allowed; in clinical trials for DMO, the majority of retreatments were administered between 4 and 7 months after the initial treatment [146]. Over the course of 3 years, patients in the Ozurdex arm of the pivotal studies received an average total of four implants [121]. In addition to DMO, Ozurdex is indicated for the treatment of macular oedema due to branch retinal vein occlusion (RVO) or central RVO and for noninfectious uveitis affecting the posterior segment of the eye [150]. It is worth noting that the use of the dexamethasone implant beyond seven implants has not been studied in a clinical trial setting [150].
 
*Iluvien (Fluocinolone Acetonide, Alimera Sciences)*
  Iluvien (190 *μ*g intravitreal implant in applicators) was first approved for its use in Australia in 2019 as a treatment for DMO in patients who were previously treated with a course of corticosteroids and did not have a clinically significant rise in IOP [151]. Its primary advantage over other corticosteroids is that the formulation allows it to remain in the eye for up to 36 months; real-world studies have shown more than one injection of Iluvien (typically at 12 months after the first implant) or additional treatment with intravitreal anti-VEGF agents is typically necessary [159]. The Fluocinolone Acetonide in Diabetic Macular Edema (FAME) A and B randomised clinical trials show the implant to be effective through month 36 [160]. These studies also showed the efficacy of the implant for chronic DMO that is not responsive to other treatments [159]. The FAME study also confirmed Iluvien's efficacy and safety in phakic patients with DMO scheduled to undergo cataract surgery [161].
 
*Triamcinolone acetonide (Multiple sources)*
  Intravitreal triamcinolone acetonide can improve vision and reduces macular thickness in the eyes with refractory DMO for up to 2 years, but repeated treatments are likely to be necessary [148]. RANZCO notes that intravitreal triamcinolone can be associated with sterile (noninfectious) endophthalmitis [162]. The use of intravitreal triamcinolone is considered off-label in Australia.

#### 7.3.1. Safety

Although three different corticosteroids are approved to treat DMO in Australia, they are not without potential safety concerns, of which ocular hypertension and cataract formation are the most significant [154, 159]. Some studies suggest that these potential adverse effects should be weighed against the benefits of a lower-cost alternative to anti-VEGF injections [163].

*(1) Cataract*. The use of corticosteroids has been associated with posterior subcapsular cataracts, and the incidence of cataracts increases after multiple corticosteroid injections [150, 154]. However, in pseudophakic patients or in those scheduled to undergo cataract surgery, higher gains in BCVA and reductions in central macular thickness than those in anti-VEGF treatments have been reported [164–166]. In the US, these concerns initially limited the use of the dexamethasone implant to pseudophakic and phakic patients scheduled for cataract surgery only, but these restrictions were later removed.

*(2) Elevated Intraocular Pressure/Glaucoma*. Ocular steroid treatment and intravitreal injections are expected to increase IOP [150, 154]. These IOP elevations (sometimes referred to as spikes) do not automatically mean that the patient has glaucoma, and not all patients with elevated IOP levels develop glaucoma [167, 168]. In the majority of cases, the elevated IOP can be managed with topical IOP-lowering medications. Furthermore, studies have found that these IOP elevations are short lived and mean that IOP often returns to the baseline between treatment cycles [169, 170].

Of the patients experiencing an increase of IOP of ≥10 mmHg from the baseline after use of the dexamethasone implant, the greatest proportion showed this IOP increase between 45 and 60 days following an injection [150]. With triamcinolone acetonide, one study showed that over a 3-year period, IOP increased by ≥10 mmHg in 33% of patients in the triamcinolone group compared with 4% in the laser arm [171].

A recent post hoc analysis of the FAME study has found that, although IOP increases were more than tripled in the fluocinolone arm (37%) than those in the placebo arm (12%), glaucomatous optic nerve changes were similar [170]. The European IRISS group supported these findings: 23% of patients in the fluocinolone arm required IOP-lowering medication but did not show clinically significant changes in cup-to-disc ratios [172]. The wide disparity between these two study results may be attributed to inclusion criteria: some patients in the IRISS study (5.2%) had baseline IOPs of >21 mmHg—an exclusion criterion for the FAME studies.

This group continues to support regular monitoring of IOP, irrespective of baseline IOP, after use of intravitreal corticosteroids and finds that immediate management is required when any elevation is noted.

*(3) Other Conditions*. There have been several reports in the literature about anterior chamber migration of the dexamethasone implant when there is communication between the posterior segment and the anterior segment [173–175]. This migration can lead to severe corneal oedema and subsequent corneal transplantation [175].

Additional safety concerns with the dexamethasone implant include the use of anticoagulants. In the pivotal studies on the dexamethasone implant, there was a similar frequency of haemorrhagic adverse events between the dexamethasone and placebo groups amongst the 8% of subjects who were on anticoagulant therapy (29% vs. 32%, respectively), but the frequency was not substantially reduced in those who did not use anticoagulant therapy (27% in the dexamethasone arm vs. 20% in the placebo arm) [150].

Haemorrhagic adverse events for those on antiplatelet medication were also reported in a slightly higher proportion of patients injected with dexamethasone implants (up to 29%) than those in the placebo group (up to 23%), irrespective of indication or the number of treatments [152]. We, therefore, recommend caution when choosing the dexamethasone implant for patients on either anticoagulant or antiplatelet medications. It is well known that systemic corticosteroids can induce central serous retinopathy. The association between dexamethasone implants and central serous retinopathy has been reported [176].

However, the stability of the dexamethasone implant allows it to be considered a first-line treatment for centre-involving DMO in various situations including “in the context of a recent arterial thromboembolic event” [177].

Intravitreal triamcinolone acetonide complications include both infectious and noninfectious inflammation (endophthalmitis/sterile endophthalmitis) and retinal detachment in addition to cataracts (both traumatic and steroid-induced) and elevated IOP [154]. RANZCO guidance recommends that eyes developing endophthalmitis post-triamcinolone injection be managed as if the eye has infectious endophthalmitis, with management strategies to involve vitreous and aqueous taps for microbiology, followed by intravitreal broad-spectrum antibiotics; prompt treatment of suspected endophthalmitis is deemed essential. RANZCO further suggests that all clinics providing intravitreal injection therapy have an endophthalmitis kit available [156].

#### 7.3.2. Efficacy

Visual and anatomic outcomes are generally better in treatment-naïve eyes than in those refractory to anti-VEGF agents. The efficacy of corticosteroids in DMO as a first-line therapy has been shown in several multinational studies, including the International Retina Group Real-Life (IRGREL) 24-month study on the dexamethasone implant. The study only enrolled 130 eyes (*N* = 125 patients); 71 eyes were treatment naïve and 59 eyes were refractory to anti-VEGF treatment. The outcome of all other eyes treated at these centres and not selected for this study was not reported. While both groups improved vision after 24 months, treatment-naïve eyes gained 11.3 ± 10.0 letters compared to 7.3 ± 2.7 letters in the refractory group [136]. Similarly, the DR-Pro-DEX study group found that the dexamethasone implant was able to delay progression of DR and PDR development while showing potential to improve DR severity in treatment-naïve eyes [178]. Others have also shown 10+ letter gains in treatment-naïve eyes [179].

Bilgic et al. [180] evaluated treatment-naïve phakic eyes with clinically significant DMO (*N* = 153) who were treated with the dexamethasone implant. At 2 years with PRN treatment, the mean CDVA improved from 0.62 to 0.4 logMAR and the median CST improved from 397 to 236 *μ*m with a median of 1.6 injections. However, as in other studies on corticosteroids, cataract development occurred (albeit in only 3 patients), and 31 patients required topical antiglaucoma therapy. Proliferative disease developed in 4 patients, which was managed with panretinal photocoagulation.

In a head-to-head comparison between dexamethasone and ranibizumab, dexamethasone implants met the noninferiority criterion in average change from baseline BCVA over 12 months; noninferiority was achieved with an average of 2.85 implant injections compared to 8.70 intravitreal ranibizumab injections. The study further showed that the percentage of patients with ≥15-letter BCVA gain from the baseline ranged from 7.2 to 17.7% with the dexamethasone implant and 4.4 to 26.9% with ranibizumab. Both the dexamethasone implant and ranibizumab effectively reduced CRT and reduced the area of fluorescein leakage. Between-group differences in change from baseline CRT favored the dexamethasone implant at 1, 2, 6, and 7 months (*P* ≤ 0.007) and ranibizumab at 4, 5, 9, and 10 months (*P* < 0.001); the decrease in the fluorescein leakage area was greater with the dexamethasone implant than with ranibizumab at month 12 (*P* < 0.001) [181].

The efficacy of corticosteroid implants in refractory eyes is well established (MEAD/CHAMPLAIN and FAMOUS/FAME studies). In MEAD, 22.2% of patients achieved ≥15 letter gain in the Ozurdex-treated group at 3 years compared with just 12% in the sham-treated group [169]. In the FAME study, eyes with chronic DMO had a better response to steroid therapy where oedema was chronic than acute; at 36 months, 34% of patients with chronic DMO in the fluocinolone acetonide arm gained 15+ letters [182].

The CHAMPLAIN study (a prospective, multicentre, open-label, 26-week study) enrolled 55 patients with refractory DMO and a history of pars plana vitrectomy (PPV). There was a statistically significant improvement in BCVA (30.4% gaining ≥10 letters at 8 weeks, a mean of 6 letters at 8 weeks, and 3 letters at 26 weeks, respectively); anatomic outcomes were equally significant [183].

A more recent meta-analysis of four randomised clinical trials (*n* = 521 eyes) found that the dexamethasone implant improved anatomic outcomes significantly better than intravitreal anti-VEGF agents but did not improve visual outcomes. The authors attributed cataract formation/progression as the cause and reiterated the general assessment that the dexamethasone implant be recommended as a first choice for select cases, such as for pseudophakic eyes, anti-VEGF-resistant eyes, or patients reluctant to receive intravitreal injections frequently [184].

There is long-termreal-world evidence on the use of fluocinolone acetonide for refractory disease in the UK, but additional treatment was frequently required [185]. Wykoff et al. looked at DR progression in patients treated with 0.2 micrograms/day of fluocinolone acetonide over 36 months [186]. Patients treated with the continuous low-dose therapy of fluocinolone acetonide had significant slowing of the progression of DR in patients with DME. In this study, 17% of patients in the fluocinolone group had progression to proliferative DR (PDR), compared to 31% in the sham-treated eyes (*P* < 0.001) [186].

The issue of when (or if) to switch patients from anti-VEGF injections to corticosteroids in eyes with DMO or DR has not yet been firmly addressed. As noted here, in refractory cases and pseudophakic eyes, corticosteroids may be of particular benefit [187]. It has been suggested that early switching from anti-VEGF produces better anatomic outcomes than late switching, but VA gains are not affected [188].

In the Early Anti-VEGF Response and Long-term Efficacy (EARLY Analysis) of the Diabetic Retinopathy Clinical Research Network (DRCR.net) Protocol I study, Dugel et al. [189] suggested that clinicians may be able to predict long-term response after the first three anti-VEGF injections, as there was a significant correlation between the average extent of oedema in the first and second 52 weeks after treatment initiation (*r* = 0.673; *P* < 0.001), and the average extent of oedema that persisted between the first and second 52 weeks after treatment initiation. Two separate studies concluded that the benefits of early switching can be maintained through 2 years; even a later switch can provide significant improvement [190, 191]. Early switching may also ensure patient well-being and may help improve patient compliance [188].

A 2019 Spanish Delphi Panel guideline suggests that switching from intravitreal anti-VEGF agents to an intravitreal dexamethasone implant should be performed “preferably after 3 injections ”; the group also supported PRN dosing strategies “as it helps prevent undertreatment” [192]. Guidelines issued in 2017 by the European Society of Retina Specialists support corticosteroid consideration in patients after 3 to 6 anti-VEGF injections [17]. The 2019 American Society of Retina Specialists (ASRS) PAT Survey also showed that a majority of clinicians would consider a steroid after a suboptimal response to 6 anti-VEGF injections [193].

Our recommendation follows suit. We recommend considering a switch from an intravitreal anti-VEGF agent to intravitreal corticosteroids after 3 to 6 anti-VEGF injections in nonresponsive or poorly responsive eyes.

*(1) Combination Therapy*. Ciulla et al. [194] suggested in 2014 that patients can receive corticosteroid implants as foundational therapy and then receive additional treatment with laser or intravitreal anti-VEGF agents as combination therapy, which may conceivably provide some synergistic benefits. A 2019 study evaluated a single-dose dexamethasone implant as an auxiliary therapy to continued intravitreal ranibizumab treatment in patients with persistent DMO and found that adding corticosteroids resulted in rapid anatomical and visual improvement in patients who responded poorly to a ranibizumab-loading dose; these improvements were maintained with continued intravitreal ranibizumab injections [135]. In the REINFORCE study, a phase 4, prospective, multicentre (18 U.S. sites), observational study of 177 patients (180 eyes; 93.8% previously treated) the dexamethasone implant was administered as monotherapy or with other DMO therapy (55%/45%, respectively). The mean maximum BCVA change from the baseline after the first three implant injections was +9.1 letters, +7.7 letters, and +7.0 letters, respectively (*P* < 0.001); 36.0% of eyes achieved 15-letter or greater BCVA improvement. The mean maximum CRT change from the baseline was −137.7 *μ*m (*P* < 0.001) [195].

DRCR.net's Protocol U [196] (40 US sites, 129 eyes) did not show a benefit of adding intravitreous dexamethasone to continued ranibizumab therapy at 24 weeks. However, a preplanned subgroup analysis showed a greater proportion of >15 letter gain in the combination therapy group and a greater improvement in central subfield thickness. Additionally, a Cochrane review identified no additional benefit for combination therapy [197].

In general, we believe that higher quality evidence does not support routinely combining intravitreal steroids and anti-VEGF therapy for DMO, but clinicians may deem this appropriate on a case-by-case basis.

### 7.4. Surgery

For patients who remain unresponsive to medical therapy, surgery may be considered to remove epiretinal membranes or vitreomacular traction that may be contributing to macular oedema [198].

#### 7.4.1. Vitrectomy

Pars plana vitrectomy (PPV) remains a viable option in the treatment regimen for DMO and plays a role, especially in the context of managing concomitant DMO and vitreomacular interface disturbance [198]. Even in the absence of the most serious complications of proliferative diabetic retinopathy (vitreous haemorrhage and retinal detachment), there is considerable evidence that vitrectomy reduces macular oedema in patients with DMO, especially in those with clinically evident signs of a taut, thickened posterior hyaloid [199–207]. Furthermore, vitrectomy has an acceptable risk profile compared with anti-VEGF therapy considering rates of major complications for each procedure [208]. Cost-effectiveness analyses of management options for DMO suggest that early vitrectomy in patients with PDR has a similar cost per quality-adjusted life year as panretinal laser photocoagulation (PRP) alone [209].

PPV with or without separation of the hyaloid, epiretinal membrane (ERM) peeling or tractional band division, intravitreal corticosteroid delivery, and or panretinal photocoagulation is indicated in the setting of diabetic eye disease with persistent or recurrent vitreous haemorrhage which makes monitoring of DMO difficult [207, 210]. Vitrectomy allows for division of tractional bands, separation of a thickened hyaloid in the context of premacula retrohyaloid haemorrhage to reduce the likelihood of retinal toxicity, delivery of more extensive panretinal photocoagulation than what can be achieved through indirect PRP in clinics, and the intravitreal delivery of anti-VEGF or corticosteroid at the time of surgery in these patients [211]. Tractional retinal detachment (TRD) involving the macula is also an important indication for vitrectomy in the context of DMO. Vitrectomy may be performed in DMO cases when laser, anti-VEGF, or intravitreal corticosteroid therapy fails to demonstrate resolution of macula oedema [212].

Benefits of vitrectomy in the management of DMO include rapid visual improvement in patients with persistent or recurrent vitreous haemorrhage or retrohyaloid haemorrhage, facilitating clinical visualisation of the retina in the context of diabetic eye disease, in particular, monitoring macular oedema [213]. Establishing rapid visualisation of the retina in diabetic eyes is important for clinical monitoring where vitreous haemorrhage—which may persist for weeks to months—precludes adequate assessment of the retina where concomitant tractional bands and proliferative retinopathy can progress undetected. Functional assessment of vision in the context of diabetic retinopathy is made more difficult as macula ischemia and oedema often confound interpretation of tests in central macula function such as with microperimetry (MAIA macular integrity assessment, CenterVue, or MP1 microperimeter, Nidek Technologies, Italy) [214–216]. In these circumstances, adequate visualisation of the retina by clearing the media through vitrectomy and the treatment of peripheral NVE aim to preserve the central macula function. The efficacy of vitrectomy in the treatment of DMO is explained by improved transvitreal oxygenation and improved growth factor diffusion away from the premacular retina in the context of ischemia caused by diabetic retinopathy [217]. Consequently, vitreous VEGF and cytokine levels have been shown to be reduced in the eyes following vitrectomy [218].

#### 7.4.2. Concurrent Vitreomacular Traction

Concurrent vitreomacular traction (VMT) or a taut, thickened posterior hyaloid (TTPH) affects segmentation accuracy of the internal limiting membrane, resulting in a more difficult interpretation of serial OCT following intravitreal injection of anti-VEGF and/or corticosteroid treatment or retinal laser for management of DMO [219]; hence, monitoring DMO resolution, persistence, or progression is more challenging [210]. In cases of VMT, separation of a thickened hyaloid with tractional bands or removal of ERM has been demonstrated to improve visual acuity and allow for resolution of DMO [220]. In patients with impaired vision, recalcitrant DMO, and a thickened posterior hyaloid, especially in the presence of vitreomacular traction, vitrectomy surgery may be required to resolve macula oedema [213]. In patients who have developed complete PVD at the macula without vitreomacular separation or persistent premacular vitreous adhesion, spontaneous resolution of DMO has been described. Persisting TTPH or the presence of ERM exerts tangential tractional forces on the macula which affects macular thickness by either inducing or exacerbating macular oedema [221]. Evidence suggests that vitrectomy in these cases to relieve the tractional forces on the macula results in a reduction of oedema and subsequent visual improvement [177, 222, 223]. There has been conflicting evidence over the past decade about whether or not vitrectomy with internal limiting membrane (ILM) peeling can improve VA in patients with DMO who have a clinically attached, but otherwise normal, posterior hyaloid [200, 224–227]. More studies have shown that vitrectomy with ILM peeling in patients without vitreoretinal interface abnormalities is a viable treatment option for recalcitrant DMO [227, 228]. Furthermore, it has been demonstrated that in patients with long-standing DMO, the earlier vitrectomy surgery is performed, the better the visual outcome [206, 229].

Our recommendation is that vitrectomy be used only after laser, intravitreal anti-VEGF agents, and intravitreal corticosteroid treatments are proved to be insufficient, and there is concurrent ERM, TTPH, or VMT contributing to macula thickening.

*(1) Summary Statement*. Australian perspective on the initial treatment of DMO.

In patients who present with non-CI-DMO, one may consider the use of focal/grid laser therapy, especially if oedema is far from the fovea centre. Once CI-DMO develops, the option to continue observing without treatment is reasonable if best-corrected vision remains at 6/7.5 or better [230].

Once a patient with CI-DMO develops a reduced vision of 20/32 (approximately 6/9) or worse, we suggest initiating treatment with 3 loading doses of either intravitreal ranibizumab or aflibercept. There is some data to suggest patients with a VA of 6/15 or worse at presentation may benefit from treatment with aflibercept [74]. However, this superiority of aflibercept over ranibizumab was only noted in the first year of treatment in DRCR Protocol T and did not extend to the second year of treatment [119]. However, in the same study, at 2 years, amongst eyes with worse baseline VA, aflibercept had superior 2-year VA outcomes to bevacizumab. Since the time our initial research was conducted, faricimab, a bi-specific antibody that binds to both VEGF-A and angiopoietin-2, has been PBS-listed and is another treatment option that may have potential for longer duration of action than existing anti-VEGF agents.

After the initial 3-loading doses, most of the authors would continue treatment by utilising a treat and extend-type regime until resolution of macula oedema is achieved. Generally, if there is absence of macula oedema (both IRF and SRF) at the clinical visit, the interval between injections would be increased by 1–2 weeks to a maximum interval of 12–16 weeks. Ideally, one would prefer absence of oedema prior to extending the intervals between injections, but trace amounts of IRF/SRF may be tolerated if vision remains good. If response is suboptimal after 3–6 injections, a switch to a different anti-VEGF agent or steroids can be considered.

In Australia, the use of dexamethasone implants is approved under the PBS system if the patient is pseudophakic or is scheduled for cataract surgery. Alternative options would be the use of intravitreal 2 or 4 mg of triamcinolone (Kenalog A-40 or Triesence). The use of intravitreal steroids would be considered especially in the presence of systemic contraindications to anti-VEGF including pregnancy or recent arterial thromboembolic events such as a recent stroke or myocardial infarction.

## Figures and Tables

**Table 1:** NHMRC recommendations for special groups [50].

Population	Screening recommendations
Children with prepubertal diabetes	Puberty
Pregnant women with diabetes	First trimester
Gestational diabetes	Only if diabetes persists postpartum
Indigenous Australians	Yearly
Non-English-speaking backgrounds	Yearly
Vision loss not attributed to DM	Yearly
Poor systemic A1c control	Yearly
Hypertension	Yearly
Hyperlipidaemia	Yearly
Renal disease	Yearly
Long duration of diabetes	Yearly

Population	Management recommendations
NPDR detected	Screen every 3–6 months
PDR/DMO detected or unexplained fall in visual acuity	Refer to ophthalmologists within 4 weeks

**Table 2:** Glucose homeostasis and diabetes mellitus [76].

		Hyperglycaemia			
		Prediabetes		Diabetes mellitus	
Type of diabetes	Normal glucose tolerance	Impaired fasting glucose or impaired glucose tolerance	Not insulin requiring	Insulin required for control	Insulin required for survival
Type 1		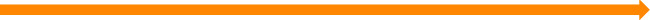			
Type 2		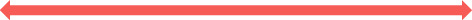			
Specific types		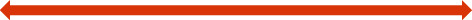			
Gestational diabetes		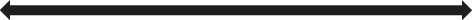			
Time (years)		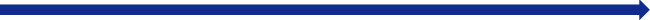			
Fasting plasma glucose	<5.6 mmol/L (100 mg/dL)	5.6–6.9 mmol/L (100–125 mg/dL)		≥7.0 mmol/L (126 mg/dL)	
2-hour plasma glucose	<7.8 mmol/L (140 mg/dL)	<7.8–11.0 mmol/L (140–199 mg/dL)		≥11.1 mmol/L (200 mg/dL)	
A1c	<5.6%	5.7–6.4%		≥6.5%	

Source: Powers et al. Harrison's principles of internal medicine, 20^th^ ed., 2018.

**Table 3:** Commonly prescribed drugs to manage DM [98].

Drug class	Examples	Mechanism of action	Side effects	Relationship with retinopathy
Biguanides (metformin) [98, 99]		Oral lowers glucose production in the liver, improves the body's sensitivity to insulin	Nausea, abdominal pain, bloating, diarrhoea	Retrospective reviews suggest that metformin may have protective effects against DR
Incretins [98, 100] (glucagon-like peptide 1 agonists)	Dulaglutide, exenatide, liraglutide, lixisenatide, semaglutide	Predominantly injection, sometimes oral mimic the action of glucagon-like peptide 1: (i) stimulate pancreatic islet *β*-cell insulin production (ii) impair glucagon secretion (iii) slow gastric emptying	Nausea, vomiting, diarrhoea, possible hypoglycaemia, but usually only when taken concurrently with other medications	May result in transient worsening of DR Case reports: (i) dramatic deterioration of DR from background retinopathy to bilateral PDR and DME (ii) rates of complications significantly higher with semaglutide
Gliptins (DPP4 inhibitors) [98, 101]	Alogliptin, linagliptin, saxagliptin, sitagliptin, vildagliptin	Oral inhibition of DPP4 (responsible for the breakdown of incretins), which enhances their action	Hypoglycaemia when taken concurrently with other medications or in combination form; upper respiratory tract infection, gastrointestinal upset, headache, skin irritation	Case reports suggest that they may reduce rates of DR progression, use of <1 year may be associated with early worsening of DR, sitagliptin showed delay and prevention of DR
Sodium glucose cotransporter-2 (SGLT2) inhibitors [98, 101]	Canagliflozin, dapagliflozin, empagliflozin, ipragliflozin	Oral tablets: reduce renal tubular glucose reabsorption, producing a reduction in blood glucose without stimulating insulin release [102]	Urinary tract infection, genital infection, hypoglycaemia	Slows progression of retinopathy in rat models, marked regression of DME after 16 weeks in case reports on ipragliflozin
Thiazolidinediones [98]	Pioglitazone, rosiglitazone	Oral: activates the gene that regulates lipid and glucose metabolism	Weight gain, fluid retention (ankle oedema), cardiac failure, bone fractures	Rosiglitazone use associated with a 59% relative risk reduction in progression to PDR over 3 years and lower rates of VA loss, may reduce inflammatory markers, and increased markers of angiogenic activity also reported the increased risk of DMO
Sulfonylureas [98, 99]	Gliclazide, glimepiride, glibenclamide, glipizide, glyburide, tolbutamide	Oral: insulin secretagogue that binds to sulfonylurea receptors in beta cells	Hypoglycaemia, weight gain	Gliclazide more so than others may prevent DR deterioration and progression to PDR and seems to have additional effects

Adapted from Saw 2019, diabetes UK, and Mayo Clinic unless otherwise cited.

## Data Availability

The references used to support the findings of this study are included within the article.

## Abbreviations

AAO: American Academy of Ophthalmology
AI: Artificial intelligence
ADA: American Diabetes Association
AMD: Age-related macular degeneration
APTOS: Asia-Pacific Tele-Ophthalmology Society
AusDiab: Australian diabetes, obesity, and lifestyle
CMO: Cystoid macular oedema
CMT: Central macular thickness
CST: Central subfoveal thickness
DCCT: Diabetes control and complications trial
DMO: Diabetic macular oedema
DR: Diabetic retinopathy
DRCR.net: Diabetic Retinopathy Clinical Research Retina Network
ESCRSS: European Society of Cataract and Refractive Surgeons
FIELD: Fenofibrate intervention and event lowering in diabetes
GPs: General practitioners
MO: Macular oedema
NEHS: National eye health study
NHMRC: National Health and Medical Research Council
NPDR: Nonproliferative diabetic retinopathy
NSAIDs: Nonsteroidal anti-inflammatory drugs
PDR: Proliferative diabetic retinopathy
POAG: Primary open-angle glaucoma
PPV: Pars plana vitrectomy
RCTs: Randomised clinical trials
RVO: Retinal vein occlusion
SD-OCT: Spectral-domain optical coherence tomography
SEED: Singapore epidemiology of eye disease
TTPH: Taut thickened posterior hyaloid
PBS: Pharmaceutical benefit scheme
VA: Visual acuity
VEGF: Vascular endothelial growth factor
WESDR: Wisconsin Epidemiologic Study of Diabetic Retinopathy
WHO: World Health Organisation.
